# RETRACTION: Ginseng and Its Active Components Ginsenosides Inhibit Adipogenesis in 3T3‐L1 Cells by Regulating MMP‐2 and MMP‐9

**DOI:** 10.1155/ecam/9786236

**Published:** 2026-02-17

**Authors:** Evidence-Based Complementary and Alternative Medicine

RETRACTION: J. Oh, H. Lee, D. Park, J. Ahn, S. S. Shin, and M. Yoon, “ Ginseng and Its Active Components Ginsenosides Inhibit Adipogenesis in 3T3‐L1 Cells by Regulating MMP‐2 and MMP‐9,” *Evidence-Based Complementary and Alternative Medicine*, 2012 (2012) https://doi.org/10.1155/2012/265023.

The above article, published online on 14 November 2012 in Wiley Online Library (https://www.wileyonlinelibrary.com), has been retracted following by John Wiley & Sons Ltd.

The retraction has been agreed following an investigation of the concerns raised by *Penicillium citreoviride* and *Actinopolyspora biskrensis* on PubPeer [1], which identified unexpected similarities between multiple panels of Figure 1. The overlapping features include the following:

- The image of 3T3‐L1 cells treated with 1 µM Rb2 appears similar to the adipocytes treated with 1 µM of Rg1.
- The cells treated with 10 µM of Rf have similar features to the adipocytes cultured with 1 µg/mL of GE.
- The image of cells treated with 10 µM of Rc appears identical to the cells treated with 10 µM of Rg3.

The authors have been contacted to comment on the abovementioned concerns and provide raw data for the images presented in the paper; however, no response was received.

Therefore, the results and integrity of this article can no longer be considered reliable.

The authors were informed of this retraction but did not provide a response.
